# Beyond the seizures: Addressing psychosocial disabilities in functional/dissociative seizures

**DOI:** 10.4102/ajod.v14i0.1682

**Published:** 2025-11-30

**Authors:** Chrisma Pretorius

**Affiliations:** 1 Department of Psychology, Faculty of Arts and Social Sciences, Stellenbosch University, Cape Town, South Africa

Disability studies, particularly in the realm of psychosocial disability, offer a critical lens through which we can understand the lived experiences of individuals navigating structural, social and psychological barriers. Prof. Leslie Swartz has been an instrumental mentor in my academic journey to excel in the field of psychosocial disability research. His guidance has shaped my academic growth and professional development in many ways. While he was not directly involved in the functional/dissociative seizures (FDS) (Hingray et al. 2025) research presented in this article, his emphasis on cultural narratives, stigma, and inclusive approaches has significantly influenced its development. Under his mentorship, I have gained a deep appreciation for the complexity of psychosocial disability, particularly in underrepresented contexts such as FDS. His ability to approach research with both intellectual rigour and humanistic empathy has been a constant source of inspiration, encouraging me to view disability not just as a medical or social issue, but as a multifaceted experience deeply rooted in individual and cultural narratives. Prof. Swartz’s mentorship extended beyond theoretical insights; he consistently emphasised the importance of critical thinking and methodological precision. His feedback on my research proposals and manuscripts was always constructive, fostering both my confidence and my ability to conduct robust, impactful research. Moreover, his encouragement to explore interdisciplinary approaches and his unwavering commitment to addressing stigma and inequality in disability contexts have influenced my research trajectory. He also exemplified the importance of mentorship itself, demonstrating how to nurture emerging researchers through patience, accessibility and a genuine investment in their success. Inspired by his approach, I have taken on a mentorship role myself, striving to nurture and empower the next generation of researchers. Through Prof. Swartz’s mentorship, I have not only refined my skills as a researcher, but have also developed a strong commitment to advancing psychosocial disability research that is inclusive, ethically grounded and culturally relevant. His mentorship has been pivotal in shaping my identity as a researcher committed to making a meaningful impact in this field.

In this article, I reflect on my research on psychosocial disability in seizure disorders, with a particular focus on the intersection between Prof. Swartz’s work on epileptic seizures (ES) and my own work on FDS.

## Distinguishing between epileptic and functional seizures

Seizure disorders are a collective way to refer to both ES and FDS, also known as psychogenic non-ES and non-epileptic attack disorder. This term encompasses both types without specifying their origin. However, it is important to note that while the term ‘seizure disorders’ is common, there can be confusion because people often associate the word ‘seizure’ strictly with ES. Differentiating FDS and ES poses a considerable clinical challenge, as both conditions often present with similar behavioural and clinical characteristics, despite having distinct underlying mechanisms, management approaches, and treatment strategies (Rawlings & Reuber 2018). In this article, starting with a clarification of the ES–FDS distinction may be useful. Epileptic seizures are caused by abnormal electrical activity in the brain, often diagnosed with Electroencephalogram (EEG) and imaging, and they respond to anti-seizure medications (Oguni 2004). Functional/dissociative seizures, on the other hand, are a physical manifestation of complex neuropsychiatric factors, often linked to stress or trauma (Popkirov et al. 2019). Functional/dissociative seizures mimic ES; but unlike the latter, they do not show abnormal brain activity on EEG (Bodde et al. 2009, 2013). Functional seizures are diagnosed through video-EEG (vEEG) monitoring, and treatment focuses on addressing underlying neuropsychiatric factors, not with seizure medications (Brown et al. 2011).

## Seizure disorders are widespread

Epilepsy is considered the most prevalent neurological disorder, with more than 80% of people with epilepsy (PWE) living in low- and middle-income countries (Owolabi et al. 2020). The overall prevalence of active epilepsy obtained in sub-Saharan Africa (SSA) (9 per 1000) is within the range of the mean value of 7.99 per 1000 population in high-income countries to 9.50 per 1000 population in low-income countries, while lifetime (16/1000) epilepsy highlights the remarkable burden of the disease in SSA (Owolabi et al. 2020). Regarding FDS, the global prevalence rate of FDS ranges from 2 to 33 per 100 000 individuals (Benbadis & Hauser 2000; Villagrán et al. 2021). Functional/dissociative seizures are more commonly observed in neurology clinics, with studies reporting FDS in 5% – 10% of outpatients and 20% – 40% of inpatients in epilepsy clinics (Asadi-Pooya & Sperling 2015). In South Africa, epidemiological research on FDS is scarce, with only one private hospital study in Johannesburg finding FDS in 50% of referred seizure patients (Anderson et al. 2017). A recent study conducted at a hospital operating under a public–private collaboration in Durban reported that 38.6% of patients with functional neurological disorders had FDS, suggesting a higher prevalence of FDS in South Africa compared with global figures (Naidoo & Bhigjee 2021). The epidemiological data clearly highlight that seizure disorders represent a significant public health concern that warrants dedicated attention and resources.

## More than epilepsy: The hidden cost of functional seizures

Seizure disorders can lead to psychosocial disability because of the potential for significant social, emotional and cognitive challenges associated with these conditions (Asadi-Pooya et al. 2021; Quintas et al. 2012). Some of these challenges include stigma, anxiety, depression, cognitive impairments and difficulties with employment and relationships, which can significantly impact a person’s quality of life and ability to function in daily activities (Asadi-Pooya et al. 2021; Quintas et al. 2012). In 2017, Swartz and colleagues conducted a review aiming to examine the literature on published studies conducted in Africa between 1994 and 2014 that examined psychosocial challenges of PWE and their carers (Keikelame et al. 2017). Several psychosocial challenges such as stigma, discrimination and marginalisation were highlighted. These challenges are compounded by cultural beliefs and a lack of understanding about epilepsy. Many PWE face barriers to employment, education and social participation, while caregivers often experience significant emotional and financial burdens. Women with epilepsy are particularly vulnerable to abuse and social exclusion. These challenges are exacerbated by limited access to epilepsy-specific healthcare, social support systems and accurate public education, underscoring the need for targeted interventions to improve the quality of life for PWE and their caregivers. In another study, Keikelame and Swartz (2018) explored the ways in which women with epilepsy (WWE) experience shame and resistance. Shame was linked to emotions such as anger, guilt, regret and grief. These emotions underscore the internal struggles WWE face as they navigate the social stigma associated with their condition. On the other hand, resistance strategies emerged as a critical theme in the stories of WWE, who described their efforts to counteract discrimination, unfair treatment and abuse – sometimes through aggressive behaviours like bullying. The study highlights that while WWE can resist societal injustices, systemic barriers such as socio-economic inequalities, marginalisation and inadequate epilepsy support services often leave them vulnerable to silent suffering and poorer health outcomes (Keikelame & Swartz 2018). It is evident that psychosocial challenges associated with epilepsy are substantial; however, those related to FDS are even greater. This is because of factors such as the pervasive lack of knowledge about the condition, frequent accusations of faking or malingering, stigma within healthcare systems, resistance to accepting the diagnosis, and limited empathy or understanding from healthcare providers (Annandale, Vilyte & Pretorius 2022; Rawlings et al. 2017, 2018). Additionally, misconceptions about the origins of FDS, societal prejudice, inadequate support systems, and the overlap with mental health conditions exacerbate the challenges faced by individuals with this disorder (Rawlings et al. 2017; Rawlings & Reuber 2016). These complexities underscore the need for contextually grounded research. I begin by reflecting on Prof. Swartz’s influence on my methodological approach, followed by an overview of research conducted in South Africa that highlights key psychosocial challenges faced by individuals with FDS and proposes strategies to address them.

## Methodological consideration and insights

Prof. Swartz’s influence on my research methodology, while not a direct application of a formal method, is evident in the choices I made to address the unique challenges of FDS in South Africa. My work intentionally moves beyond a purely clinical perspective by focusing on the psychosocial dimensions of FDS, which align with his emphasis on cultural narratives, stigma, and inclusive, ethically grounded research in the Global South (Keikelame & Swartz 2019). Rather than relying solely on quantitative data, most of my research consists of qualitative local studies to understand the lived experiences of individuals with FDS, consistent with Swartz’s call for humanistic, contextually rooted inquiry (Keikelame & Swartz 2019). Furthermore, my decision to propose holistic, locally relevant interventions that integrate medical, psychological and social care mirrors his focus on developing research that is relevant and guides best practice in a South African context. This approach, guided by his mentorship, demonstrates a commitment to advancing research that is ethically grounded and culturally relevant. This influence also extends to how I mentor scholars, cultivating a new generation of researchers who are thoughtful, reflexive, critical and socially responsive.

### Stigma, misunderstanding and social isolation

Stigma is a significant issue in FDS and serves as an underlying factor across most themes. Misunderstanding and stigma surrounding FDS often result in strained relationships and reduced social support. Social stigma from family and community members contributes to feelings of isolation and distress (Pretorius & Sparrow 2015). Individuals with FDS face stigma from healthcare providers who may perceive their condition as less legitimate than ES, leading to delayed or inadequate care (Samuels & Pretorius 2023). People with FDS may feel judged or dismissed by peers, family or even healthcare providers because of the nature of the condition (Pretorius 2016). Avoidance coping strategies are commonly used to navigate the stigma associated with FDS (Cronje & Pretorius 2013). The fear of experiencing seizures in public often leads to social withdrawal, further exacerbating isolation (Pretorius & Sparrow 2015).

### Healthcare system challenges

The South African healthcare system lacks resources, including specialists trained to diagnose and treat FDS effectively (Pretorius 2016; Vilyte, Butler & Pretorius 2023). Functional/dissociative seizures are frequently misdiagnosed as epilepsy, leading to inappropriate treatment, including unnecessary use of anti-seizure medication and delayed access to psychological interventions. People with FDS often experience fragmented care, with a lack of coordination between neurological and psychiatric services. While vEEG remains the gold standard for diagnosing FDS, it is costly and often inaccessible in South Africa, and leads to a delay in diagnosis. To address this, a machine learning-based clinical decision aid, the Functional Seizure Clinical Decision Aid – Public Healthcare (FSAid-PH), was recently developed and preliminarily validated in South Africa to support early, cost-effective identification of FDS in public healthcare (Vilyte 2024). The delay in obtaining a correct diagnosis (average of 7.2 years) creates uncertainty, leading to emotional distress and mistrust in the medical system (Pretorius 2016; Pretorius & Sparrow 2015). Although appropriate communication of the diagnosis improves treatment adherence and reduces distress, healthcare professionals struggle with how to communicate the diagnosis. This leads to confusion, frustration and poor treatment adherence (Fouché, Hartwig & Pretorius 2019; Hartwig & Pretorius 2019). Ultimately, most of these challenges stem from a lack of knowledge and education among healthcare professionals, highlighting the urgent need for improved training and awareness (Hartwig & Pretorius 2019; Pretorius 2016; Pretorius & Sparrow 2015; Samuels & Pretorius 2023).

### Employment and socioeconomic stressors

Many people with FDS face barriers in employment, either because of discrimination or because their condition prevents them from maintaining stable jobs (Samuels & Pretorius 2023). The unpredictable nature of FDS can disrupt productivity and attendance, leading to difficulty maintaining employment or completing education. Workplace discrimination and a lack of accommodations often add to the challenges. Socioeconomic stressors such as financial insecurity and a lack of access to healthcare exacerbate mental health issues (Pretorius 2016; Vilyte et al. 2023). Furthermore, people with FDS from public hospitals, generally coming from lower socioeconomic backgrounds, experience significantly more violence, financial insecurity, and a lack of access to healthcare compared with those in private hospitals (Vilyte et al. 2023).

### Dependency and family dynamics

Families often struggle to understand FDS, leading to overprotectiveness or frustration which can limit the patient’s autonomy (Pretorius & Sparrow 2015). Functional/dissociative seizures may lead to increased dependency on caregivers, disrupting family dynamics and creating financial and emotional burdens (Pretorius 2016). Marital and familial relationships are often strained, with some patients reporting partner abandonment or domestic violence following their diagnosis (Vilyte et al. 2023).

### Emotional and psychological challenges

A strong association exists between FDS and psychological trauma, particularly childhood abuse, intimate partner violence, and loss of a loved one (Vilyte et al. 2023). High rates of comorbid mental health conditions such as anxiety, depression, and post-traumatic stress disorder (PTSD), and other psychiatric comorbidities, which often go untreated, can exacerbate distress (Vilyte & Pretorius 2019). Fear and uncertainty about seizure episodes can lead to avoidance behaviours and heightened emotional instability. People with FDS tend to use avoidance coping strategies such as denial, escape, and distancing, which negatively affect their quality of life (Cronje & Pretorius 2013).

## Discussion

Functional/dissociative seizures significantly impact daily living, often leading to functional disability that makes it difficult for individuals to work, drive or engage in social activities (Pretorius & Sparrow 2015). The South African context presents significant psychosocial challenges for FDS patients, including stigma, trauma, economic hardship, and inadequate healthcare access, all of which contribute to psychosocial disability. Addressing these challenges requires the integration of medical, psychological and social interventions to improve quality of life and reduce functional impairment (Asadi-Pooya et al. 2021). At the core of these challenges is stigma, which underlies all aspects of the FDS experience – affecting diagnosis, treatment access, healthcare interactions, employment, and social relationships (Annandale et al. 2022; Hingray et al. 2018). Healthcare professionals, families and communities often misunderstand FDS, leading to marginalisation, disbelief and inadequate care. Stigma reduction must be a central focus of interventions, requiring education, advocacy and systemic change to ensure that FDS is recognised as a legitimate medical condition rather than a sign of malingering or attention-seeking behaviour (Samuels & Pretorius 2023).

Early and accurate diagnosis of FDS is essential to improving patient outcomes and reducing psychosocial disability (Doss & La France 2016). However, diagnostic delays remain a major challenge, often leaving patients misdiagnosed, untreated, or subjected to unnecessary medical interventions (Doss & La France 2016). While vEEG remains the diagnostic gold standard for FDS, its high cost and limited availability in low-resource settings present major barriers to timely diagnosis (Vilyte & Pretorius 2019). The development of machine learning-based tools like FSAid-PH offers a promising, context-sensitive solution by enabling earlier, more accessible screening, but further validation is needed to confirm its broader clinical utility (Vilyte 2024).

Beyond early diagnosis, one of the most critical steps in reducing psychosocial disability in FDS is improving knowledge, education and awareness among healthcare professionals (Hingray et al. 2018). Many providers struggle to diagnose and manage FDS because of limited training and understanding, often reinforcing stigma through dismissive attitudes or inappropriate medical treatments (Hingray et al. 2018; Samuels & Pretorius 2023). Education initiatives should focus on enhancing the recognition, diagnosis, and communication of FDS, ensuring that healthcare providers convey the legitimacy of the condition, address patient concerns with empathy, and facilitate access to appropriate interventions (Fouché et al. 2019). A multidisciplinary approach is essential, integrating neurology, psychiatry, psychology, and social work to address the complex needs of FDS patients (Fouché et al. 2019). This collaborative model allows for a holistic and coordinated care plan.

In addition to improving healthcare systems, a key element of improving outcomes for individuals with FDS is psychoeducation for both patients and their families. Providing clear, culturally relevant education on the nature of FDS can reduce fear, misinformation, and stigma while empowering patients to take an active role in managing their condition (Pretorius & Sparrow 2015). Globally, psychological therapy is regarded as the preferred treatment for the disorder, even though professionals often face significant challenges in providing it (Hingray et al. 2018). In addition, addressing comorbidities is crucial, as depression, anxiety, PTSD, and personality disorders frequently co-occur with FDS and exacerbate functional impairment (Vilyte & Pretorius 2019). Effective treatment of these comorbidities – through integrated psychiatric and psychological interventions – can significantly improve both seizure outcomes and overall well-being.

While social isolation is often considered a significant issue for individuals with FDS, a study aimed at exploring the socialisation characteristics of people with FDS indicated substantial engagement in social activities (Vaidya-Mathur et al. 2016). This suggests that socialisation patterns may be more complex and nuanced than initially assumed. The most frequently cited barriers to socialisation were driving restrictions and medication side effects. When asked about preferred support options, respondents showed the highest interest in online support groups or educational programmes (29.46%), followed by office-based support groups (28.57%) and volunteering opportunities (23.21%). Future therapeutic interventions should therefore consider offering both remote and in-person support options tailored to individual preferences and age-related needs (Vaidya-Mathur et al. 2016). In addition, a key treatment goal should focus on improving employment opportunities and providing job training for individuals with FDS who can work (Vaidya-Mathur et al. 2016). To address work-related challenges, collaboration with employers to increase awareness and understanding of FDS can help reduce workplace discrimination and create a more inclusive work environment.

## Conclusion

To effectively address the multifaceted psychosocial burden of FDS, interventions must be comprehensive, integrating education, multidisciplinary care, therapy, and social support. However, these interventions must be grounded in research that is locally relevant and attuned to the unique sociocultural and systemic challenges of the South African context. While people with FDS may share some difficulties with other disability groups, they face a distinct set of challenges: persistent stigma from healthcare providers and communities, frequent accusations of malingering or attention-seeking, prolonged delays in diagnosis, and difficulty accessing appropriate psychological care because of the fragmented nature of services. The invisibility and contested legitimacy of the condition often result in emotional distress, social isolation, and a loss of autonomy. These complexities demand solutions that are not only clinically appropriate, but also sensitive to the structural inequalities and cultural dynamics within which FDS is experienced. Addressing these challenges requires not only context-specific knowledge production, but also the cultivation of a new generation of scholars equipped to engage with these realities. Drawing on the example of strong academic mentorship, such as that provided by Prof. Leslie Swartz, this work underscores the critical role that mentorship plays in shaping methodologically rigorous, socially responsive, and ethically grounded research. Strengthening both research and clinical practice in South Africa depends on sustained mentorship, interdisciplinary collaboration, and a continued commitment to producing solutions that are generated within and for the local context.

## References

[CIT0001] Anderson, D.G., Damianova, M., Hanekom, S. & Lucas, M., 2017, ‘A comparative retrospective exploration of the profiles of patients in South Africa diagnosed with epileptic and psychogenic non-epileptic seizures’, *Epilepsy & Behavior* 69, 37–43. 10.1016/j.yebeh.2017.01.005 28222340

[CIT0002] Annandale, M., Vilyte, G. & Pretorius, C., 2022, ‘Stigma in functional seizures: A scoping review’, *Seizure* 99, 131–152. 10.1016/j.seizure.2022.05.016 35640468

[CIT0003] Asadi-Pooya, A.A., Brigo, F., Kozlowska, K., Perez, D.L., Pretorius, C., Sawchuk, T. et al., 2021, ‘Social aspects of life in patients with functional seizures: Closing the gap in the biopsychosocial formulation’, *Epilepsy & Behavior* 117, 107903. 10.1016/j.yebeh.2021.107903 33740497

[CIT0004] Asadi-Pooya, A.A. & Sperling, M.R., 2015, ‘Epidemiology of psychogenic nonepileptic seizures’, *Epilepsy & Behavior* 46, 60–65. 10.1016/j.yebeh.2015.03.015 25882323

[CIT0005] Benbadis, S.R. & Hauser, W.A., 2000, ‘An estimate of the prevalence of psychogenic non-epileptic seizures’, *Seizure* 9, 280–281. 10.1053/seiz.2000.0409 10880289

[CIT0006] Bodde, N.M.G., Brooks, J.L., Baker, G.A., Boon, P.A.J.M., Hendriksen, J.G.M., Mulder, O.G. et al., 2009, ‘Psychogenic non-epileptic seizures-Definition, etiology, treatment and prognostic issues: A critical review’, *Seizure* 18, 543–553. 10.1016/j.seizure.2009.06.006 19682927

[CIT0007] Bodde, N.M.G., Van der Kruijs, S.J.M., Ijff, D.M., Lazeron, R.H.C., Vonck, K.E.J. & Boon, P.A.J.M., 2013, ‘Subgroup classification in patients with psychogenic non-epileptic seizures’, *Epilepsy & Behavior* 26, 279–289. 10.1016/j.yebeh.2012.10.012 23200772

[CIT0008] Brown, R.J., Syed, T.U., Benbadis, S., LaFrance, Jr, W.C. & Reuber, M., 2011, ‘Psychogenic nonepileptic seizures’, *Epilepsy & Behavior* 22(1), 85–93. 10.1016/j.yebeh.2011.02.016 21450534

[CIT0009] Cronje, G. & Pretorius, C., 2013, ‘The coping styles and health-related quality of life of South African psychogenic non-epileptic seizures patients’, *Epilepsy & Behavior* 29(3), 581–584. 10.1016/j.yebeh.2013.09.045 24169204

[CIT0010] Doss, R.C. & LaFrance, W.C., Jr., 2016, ‘Psychogenic non-epileptic seizures’, *Epileptic Disorders* 18, 337–343. 10.1684/epd.2016.0873 27818368

[CIT0011] Fouché, M., Hartwig, L. & Pretorius, C., 2019, ‘Management of uncertainty in the diagnosis communication of psychogenic non-epileptic seizures in a South African context’, *Epilepsy & Behavior* 98, 45–52. 10.1016/j.yebeh.2019.06.009 31299532

[CIT0012] Hartwig, L. & Pretorius, C., 2019, ‘Psychogenic non-epileptic seizures: Comparing what South African healthcare providers communicate to patients at the point of diagnosis against international guidelines’, *Epilepsy & Behavior* 101, 106399. 10.1016/j.yebeh.2019.06.042 31698264

[CIT0013] Hingray, C., El-Hage, W., Duncan, R., Gigineishvili, D., Kanemoto, K., LaFrance, W.C. et al., 2018, ‘Access to diagnostic and therapeutic facilities for psychogenic non-epileptic seizures: An international survey by the ILAE PNES Task Force’, *Epilepsia* 59(1), 203–214. 10.1111/epi.13952 29152734

[CIT0014] Hingray, C., Popkirov, S., Kozlowska, K., Pretorius, C., Sarudiansky, M., El-Hage, W. et al., 2025, ‘Functional/dissociative seizures: Proposal for a new diagnostic label and definition by the ILAE task force’, *Epilepsia* 1–21. 10.1111/epi.18574 PMC12661283 40884444

[CIT0015] Keikelame, M.J., Suliaman, T., Hendriksz, M. & Swartz, L., 2017, ‘Psychosocial challenges affecting the quality of life in adults with epilepsy and their carers in Africa: A review of published evidence between 1994 and 2014’, *African Journal of Primary Health Care & Family Medicine* 9(1), a1275. 10.4102/phcfm.v9i1.1275 PMC5387367 28397523

[CIT0016] Keikelame, M.J. & Swartz, L., 2018, ‘“I wonder if I did not mess up….”: Shame and resistance among women with epilepsy in Cape Town, South Africa’, *Seizure: European Journal of Epilepsy* 61, 50–56. 10.1016/j.seizure.2018.07.021 30096624

[CIT0017] Keikelame, M.J. & Swartz, L., 2019, ‘Decolonising research methodologies: Lessons from a qualitative research project, Cape Town, South Africa’, *Global Health Action* 12(1), 1561175. 10.1080/16549716.2018.1561175 30661464 PMC6346712

[CIT0018] Naidoo, L. & Bhigjee, A.I., 2021, ‘The spectrum of functional neurological disorders: A retrospective analysis at a tertiary hospital in South Africa’, *South African Journal of Psychiatry* 27, 1–9. 10.4102/sajpsychiatry.v27i0.1607 PMC8063761 33936802

[CIT0019] Oguni, H., 2004, ‘Diagnosis and treatment of epilepsy’, *Epilepsia* 45(Suppl. 8), 13–16. 10.1111/j.0013-9580.2004.458003.x 15610188

[CIT0020] Owolabi, L.F., Adamu, B., Jibo, A.M., Owolabi, S.D., Isa, A.I., Alhaji, I.D. et al., 2020, ‘Prevalence of active epilepsy, lifetime epilepsy prevalence, and burden of epilepsy in sub-Saharan Africa from meta-analysis of door-to-door population-based surveys’, *Epilepsy & Behavior* 103, 106846. 10.1016/j.yebeh.2019.106846 31941583

[CIT0021] Popkirov, S., Asadi-Pooya, A.A., Duncan, R., Gigineishvili, D., Hingray, C., Kanner, A.M. et al., 2019, ‘The aetiology of psychogenic non-epileptic seizures: Risk factors and comorbidities’, *Epileptic Disorders* 21(6), 529–547. 10.1684/epd.2019.1107 31843732

[CIT0022] Pretorius, C., 2016, ‘Barriers and facilitators to reaching a diagnosis of PNES from the patients’ perspective: Preliminary findings’, *Seizure* 38, 1–6. 10.1016/j.seizure.2016.03.007 27039015

[CIT0023] Pretorius, C. & Sparrow, M., 2015, ‘Life after being diagnosed with psychogenic non-epileptic seizures (PNES): A South African perspective’, *Seizure* 30, 32–41. 10.1016/j.seizure.2015.05.008 26216682

[CIT0024] Quintas, R., Raggi, A., Giovannetti, A.M., Pagani, M., Sabariego, C., Cieza, A. et al., 2012, ‘Psychosocial difficulties in people with epilepsy: A systematic review of literature from 2005 until 2010’, *Epilepsy & Behavior* 25(1), 60–67. 10.1016/j.yebeh.2012.05.016 22749606

[CIT0025] Rawlings, G.H., Brown, I., Stone, B. & Reuber, M., 2017, ‘Written accounts of living with psychogenic nonepileptic seizures: A thematic analysis’, *Seizure* 50, 83–91. 10.1016/j.seizure.2017.06.006 28633044

[CIT0026] Rawlings, G.H., Brown, I., Stone, B. & Reuber, M., 2018, ‘Written accounts of living with epilepsy or psychogenic nonepileptic seizures: A thematic comparison’, *Qualitative Health Research* 28(6), 950–962. 10.1177/1049732317748897 29291685

[CIT0027] Rawlings, G.H. & Reuber, M., 2016, ‘What patients say about living with psychogenic nonepileptic seizures: A systematic synthesis of qualitative studies’, *Seizure* 41, 100–111. 10.1016/j.seizure.2016.07.014 27522576

[CIT0028] Samuels, T. & Pretorius, C., 2023, ‘Healthcare providers’ perspectives on stigma when working with people with functional seizures’, *Seizure: European Journal of Epilepsy* 112, 121–127. 10.1016/j.seizure.2023.10.002 37820427

[CIT0029] Vaidya-Mathur, U., Myers, L., Laban-Grant, O., Lancman, M., Lancman, M. & Jones, J., 2016, ‘Socialization characteristics in patients with psychogenic nonepileptic seizures (PNES)’, *Epilepsy & Behavior* 56, 59–65. 10.1016/j.yebeh.2015.12.032 26844647

[CIT0030] Villagrán, A., Eldøen, G., Duncan, R., Aaberg, K.M., Hofoss, D. & Lossius, M.I., 2021, ‘Incidence and prevalence of psychogenic nonepileptic seizures in a Norwegian county: A 10-year population-based study’, *Epilepsia* 62(7), 1528–1535. 10.1111/epi.16949 34075579

[CIT0031] Vilyte, G., 2024, ‘Psychogenic non-epileptic seizure (PNES) patient profiles in two Western Cape epilepsy monitoring units and their utility in PNES diagnosis’, Doctoral dissertation, Stellenbosch University, Stellenbosch University Research Repository.

[CIT0032] Vilyte, G., Butler, J. & Pretorius, C., 2023, ‘Prevalence and comparison of psychological trauma and stressors in functional seizure patients from a public and private hospital’, *Seizure: European Journal of Epilepsy* 112, 112–120. 10.1016/j.seizure.2023.10.001 37797429

[CIT0033] Vilyte, G. & Pretorius, C., 2019, ‘Personality traits, illness behaviours and psychiatric comorbidity in individuals with psychogenic non-epileptic seizures (PNES), epilepsy and other non-epileptic seizures (oNES): Differentiating between the conditions’, *Epilepsy & Behavior* 98(Part A), 210–219. 10.1016/j.yebeh.2019.05.043 31382179

